# Supplementary material for: Modeling glioblastoma heterogeneity as a dynamic network of cell states
Source: Mol Syst Biol. 2021 Sep 16;17(9):e10105. doi: 10.15252/msb.202010105 (PMC8444284; doi:10.15252/msb.202010105)
Supplement: Supplementary file 5 — Source Data for Figure 3 [file MSB-17-e10105-s001.zip › Figure3A_sourcedata/GSEA_3065/hallmarks_state3.GseaPreranked.1623416274744/HALLMARK_HEDGEHOG_SIGNALING.html]

Details for gene set HALLMARK\_HEDGEHOG\_SIGNALING[GSEA]

|  || Dataset | state3 |
| Phenotype | NoPhenotypeAvailable |
| Upregulated in class | na\_pos |
| GeneSet | HALLMARK\_HEDGEHOG\_SIGNALING |
| Enrichment Score (ES) | 0.48600692 |
| Normalized Enrichment Score (NES) | 1.4041632 |
| Nominal p-value | 0.08409091 |
| FDR q-value | 0.090298295 |
| FWER p-Value | 0.416 |
Table: GSEA Results Summary

  

Fig 1: Enrichment plot: HALLMARK\_HEDGEHOG\_SIGNALING      
 Profile of the Running ES Score & Positions of GeneSet Members on the Rank Ordered List

  

| PROBE | GENE SYMBOL | GENE\_TITLE | RANK IN GENE LIST | RANK METRIC SCORE | RUNNING ES | CORE ENRICHMENT || 1 | ADGRG1 |  |  | 168 | 0.301 | 0.1117 | Yes |
| 2 | THY1 |  |  | 326 | 0.236 | 0.1964 | Yes |
| 3 | NF1 |  |  | 425 | 0.214 | 0.2780 | Yes |
| 4 | MYH9 |  |  | 688 | 0.173 | 0.3231 | Yes |
| 5 | PTCH1 |  |  | 743 | 0.165 | 0.3887 | Yes |
| 6 | SCG2 |  |  | 778 | 0.160 | 0.4543 | Yes |
| 7 | AMOT |  |  | 1022 | 0.137 | 0.4860 | Yes |
| 8 | VEGFA |  |  | 1723 | 0.090 | 0.4451 | No |
| 9 | OPHN1 |  |  | 1858 | 0.083 | 0.4658 | No |
| 10 | VLDLR |  |  | 2734 | 0.049 | 0.3869 | No |
| 11 | TLE3 |  |  | 3709 | 0.022 | 0.2851 | No |
| 12 | NRCAM |  |  | 4278 | 0.007 | 0.2232 | No |
| 13 | PML |  |  | 4353 | 0.005 | 0.2170 | No |
| 14 | RASA1 |  |  | 4447 | 0.003 | 0.2075 | No |
| 15 | CELSR1 |  |  | 4969 | -0.010 | 0.1522 | No |
| 16 | NRP2 |  |  | 5175 | -0.015 | 0.1353 | No |
| 17 | ETS2 |  |  | 5490 | -0.024 | 0.1097 | No |
| 18 | CRMP1 |  |  | 6243 | -0.048 | 0.0445 | No |
| 19 | LDB1 |  |  | 6336 | -0.051 | 0.0559 | No |
| 20 | HEY1 |  |  | 6933 | -0.077 | 0.0213 | No |
| 21 | DPYSL2 |  |  | 7583 | -0.126 | 0.0016 | No |
| 22 | CDK6 |  |  | 7605 | -0.128 | 0.0548 | No |
| 23 | TLE1 |  |  | 7945 | -0.176 | 0.0923 | No |
Table: GSEA details [plain text format]

  

Fig 2: HALLMARK\_HEDGEHOG\_SIGNALING: Random ES distribution      
 Gene set null distribution of ES for **HALLMARK\_HEDGEHOG\_SIGNALING**
